# The baton changed hands

**DOI:** 10.1186/s10194-021-01288-6

**Published:** 2021-08-03

**Authors:** Paolo Martelletti

**Affiliations:** 1 Department of Clinical and Molecular Medicine, Sapienza University, Rome, Italy; 2 Sant’Andrea University Hospital, Via di Grottarossa 1035, 00189 Rome, Italy

**Keywords:** Indexing, Impact factor, Journal Citation Reports, Clarivate Analytics

“Omnium rerum principia parva sunt [1].”

On the 30th of June 2021, Clarivate Analytics announced the release of 2020 Journal Citation Reports (JCR), announcing the 2020 Impact Factor (IF) of *The Journal of Headache and Pain* as 7.277. This represents a big leap ahead (+ 52%) compared to the IF of 2019: 4.797, and it is the highest IF ever recorded for a headache journal [2]. This places *The Journal of Headache and Pain* as the first headache journal, in the first quarter (Q1) for the category of Neurosciences—where the journal now ranks 36th out of 273 journals—and in the Q1 for the category Clinical Neurology (where it ranks 20th out of 208 journals).

We are aware that this year Clarivate changed how it calculates IFs, and this will have had some positive effect. This year’s IF traditionally would have considered citations in 2020 for content that was published in issues in 2018 and 2019. This year Clarivate adjusted the ‘cut-off’ point for inclusion of content, from the issue date to the date of online publication for content published from 2020, thereby increasing the number of considered citations in the new IF calculation (the number of articles from which citations are collected will be higher; the IF denominator is unchanged) [3]. While this factor may give some positive push, it alone cannot be responsible for the huge leap we have made.

All credit goes to the Co-Editors, Associate Editors, Advisory Board, and Junior Editorial Board and also to our Reviewers and Authors for the choice of content that has been published in our journal and for their contribution to the journal growth since its foundation [4]. Thank you to the journal-affiliated societies, the European Headache Federation (EHF) and Lifting the Burden, for their trust over so many years.

We are deeply grateful for the help and support of the whole team, and we are proud of this important and “unbelievable” achievement that we owe to them. However, although many have used the term “unbelievable,” I prefer to say “ensuing” global success. This achievement is a result of a tight and continuous team working together, sometimes sailing upwind, close-hauled.

Of course, citation is not the only measure for high impact; thus, I am more than happy to acknowledge that the journal has been growing over the years in quality, volume, and reach as well. Submissions increased by 28% in 2020 vs 2019 (426 submissions in 2020) and are peer-reviewed with excellent turnaround times: on average, it takes 17 days from submission to first decision and 68 days from submission to acceptance. Publications have increased over the years, and in 2020, 136 articles were published in the journal (+ 20% vs 2019). Full-text downloads increased astoundingly in 2020 (approximately 1,531,418 downloads, + 69% vs 2019) along with the number of shares on social media.

Much is also due to all those who said many years ago that a new journal in the field of headaches “could not be done” and that “it should not have been done,” and we also owe a lot to those who said that “they would have never applied for an article processing charge”—enough to understand that we had to continue developing the project.

A multivoiced scientific community is more authoritative, and it tends to compete fairly and improve itself.

It is also interesting to note how once again all the headache journals have increased their IFs, witnessing the moment of good health that our scientific community is experiencing. We are no longer considered as the “Cinderella of neuroscience.” Of course, a significant boost to this cultural growth can be attributed to the new classes of dedicated drugs that are still struggling but progressively permeating migraine therapy. Certainly, another impetus is given by the enormous amount of educational activity carried out in recent years by scientific societies in the training of new recruits, who are already showing their scientific skills as promising rookies.

## The baton changed hands

We know that there are no immutable situations, even if guaranteed by mandatory citability privileges, and we will work to consolidate this result, opening ourselves to new ideas and connecting with advanced areas of biomedical research in headaches with our open access contents, without leaving out emerging areas. Our perspective will continue to be multidisciplinary, given that headaches are a topic of study from different perspectives and specialties; scientifically, they are not a privilege of the few. Young people will continue to receive easy access through successful initiatives such as the EHF School of Advanced Studies Reviews.

However, we continue to believe that headaches must also be clinically tackled on the vast platform of clinical medicine, and for this reason we tend to operate on multiple fronts.

We are fortunate to have on our side a publisher who is rigorous on quality, prone to new ideas, striving toward innovations, anchored to best practices in publishing, and in any case always respectful of the scientific choices made in the selection of the works to be promoted.

The projects in the pipeline are many. Several thematic series are still open for submissions, such as the one on Translational Research in Headache and Basic Science in Headache. A thematic series has just been opened to collect all papers that over time won the Enrico Greppi Award, traditionally hosted here. New exciting Thematic Series will be launched soon.

A refreshment of the Editorial Board in the forthcoming months will allow us to welcome those who volunteered to join our team to help us build a competitive, fast, independent “headache-research world” that is constantly renewed in quality and dedicated to what we have chosen over time, the care of headache sufferers.
